# Extracting the abstraction pyramid from complex networks

**DOI:** 10.1186/1471-2105-11-411

**Published:** 2010-08-03

**Authors:** Chia-Ying Cheng, Yuh-Jyh Hu

**Affiliations:** 1 Department of Computer Science, National Chiao Tung University, 1001 University Rd. Hsinchu, Taiwan; 2 Institute of Biomedical Engineering, National Chiao Tung University, 1001 University Rd. Hsinchu, Taiwan

## Abstract

**Background:**

At present, the organization of system modules is typically limited to either a multilevel hierarchy that describes the "vertical" relationships between modules at different levels (e.g., module A at level two is included in module B at level one), or a single-level graph that represents the "horizontal" relationships among modules (e.g., genetic interactions between module A and module B). Both types of organizations fail to provide a broader and deeper view of the complex systems that arise from an integration of vertical and horizontal relationships.

**Results:**

We propose a complex network analysis tool, Pyramabs, which was developed to integrate vertical and horizontal relationships and extract information at various granularities to create a pyramid from a complex system of interacting objects. The pyramid depicts the nested structure implied in a complex system, and shows the vertical relationships between abstract networks at different levels. In addition, at each level the abstract network of modules, which are connected by weighted links, represents the modules' horizontal relationships. We first tested Pyramabs on hierarchical random networks to verify its ability to find the module organization pre-embedded in the networks. We later tested it on a protein-protein interaction (PPI) network and a metabolic network. According to Gene Ontology (GO) and the Kyoto Encyclopedia of Genes and Genomes (KEGG), the vertical relationships identified from the PPI and metabolic pathways correctly characterized the *inclusion *(i.e., *part-of*) relationship, and the horizontal relationships provided a good indication of the functional closeness between modules. Our experiments with Pyramabs demonstrated its ability to perform knowledge mining in complex systems.

**Conclusions:**

Networks are a flexible and convenient method of representing interactions in a complex system, and an increasing amount of information in real-world situations is described by complex networks. We considered the analysis of a complex network as an iterative process for extracting meaningful information at multiple granularities from a system of interacting objects. The quality of the interpretation of the networks depends on the completeness and expressiveness of the extracted knowledge representations. Pyramabs was designed to interpret a complex network through a disclosure of a pyramid of abstractions. The abstraction pyramid is a new knowledge representation that combines vertical and horizontal viewpoints at different degrees of abstraction. Interpretations in this form are more accurate and more meaningful than multilevel dendrograms or single-level graphs. Pyramabs can be accessed at http://140.113.166.165/pyramabs.php/.

## Background

Networks provide a natural representation for the complex interactions of heterogeneous entities in complex systems. Many complex networks have been studied in recent years, for example in the fields of biology, sociology, and ecology [1-4]. As high-throughput techniques have advanced, biological networks have become increasingly complex, and it has become more challenging to interpret them accurately and clearly by extracting and representing the knowledge embedded in the networks.

**Figure 1:**
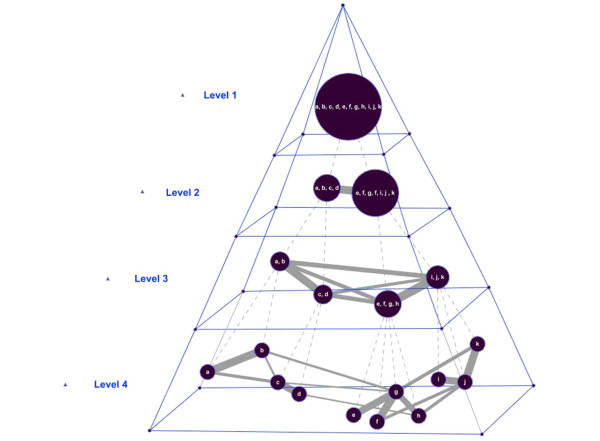
**Illustration of vertical and horizontal relationships**. Each circle represents a module. Vertical relationships and horizontal relationships are denoted by dashed lines and solid lines, respectively. The thickness of a solid line increases with the importance of the connection. The original network is at the bottom (Level 4). Higher-level networks are an abstraction, to a certain degree, of the next lowest network.

The concept of modularity has a long history in biology; for example, it has been proposed that biological processes within individual cells are modular [5,6]. Module-level studies have accelerated the progress of system biology, e.g., modular organizations of cellular networks, module-level analyses in gene networks, and modular network models of aging [7-9]. One common approach to mining complex networks based on modularity is first to identify modules as knowledge building blocks, and then to use their organization to depict the knowledge contained in the networks. However, most module organizations are limited to either a "vertical" or "horizontal" representation. A vertical relationship is represented by a multilevel dendrogram that only describes the *inclusion*/*part-of *relationships between modules at different hierarchical levels [4,10,11], and the horizontal relationship is a single-level graph that only shows how modules are connected [7-9]. Neither of them provides an integrated view of the complex systems they represent; consequently, it is difficult to further explore these complex domains. In this work, we combine vertical and horizontal relationships in order to organize the modules into a multilevel pyramid, as illustrated in Figure 1. At each level, we describe the horizontal relationships by a network of modules that is by itself the abstraction of the network at a lower level [3]. In contrast, the vertical relationships, shown as links between layers, represent the *inclusion *relationship between modules at different levels. Using an abstraction pyramid, not only can domain experts gain a global multilevel view of a complex system from two different perspectives (horizontal and vertical), but they can also investigate the interconnection of the modules at a particular abstraction level of interest in the hierarchy.

Our approach, named Pyramabs (Pyramid of abstractions), identifies the modules and simultaneously constructs the pyramid based on the network topology. Prior domain knowledge is not used. We tested Pyramabs on artificial random networks, a protein-protein interaction network, and a metabolic network. We compared Pyramabs with other methods and verified our results based on those published in the literature and public databases.

## Results

The two overarching goals of our work are to (1) propose an alternative knowledge representation for improved network interpretations, and (2) introduce a novel approach for extracting knowledge from networks and describing it using the new representation. The abstraction pyramid discovered by Pyramabs does not replace the known structure of ontology (e.g., the Gene Ontology (GO)), but instead provides other information that may be missing. For example, an abstraction pyramid identified from a protein-protein interaction network could illuminate the protein interactions at various levels. Some vertical or horizontal relationships can provide additional biological meaning that may not be characterized in the GO's Directed Acyclic Graph (DAG) structure.

**Figure 2:**
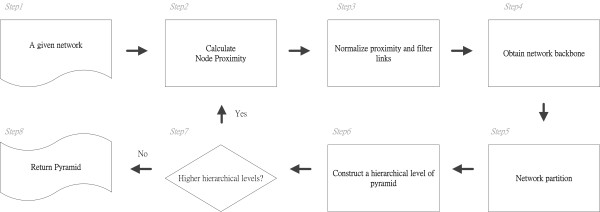
**System Flow of Pyramabs**.

We divide the analysis of complex networks into two tasks: module discovery and module organization. The novelty of our two-way approach is derived from the synergy of top-down and bottom-up clustering algorithms. This method identifies modules in a top-down fashion and constructs a hierarchy implied in a complex network from the bottom up. In addition, it produces an abstraction of the network to different degrees at different levels in the hierarchy. Our method can be divided into three procedures: (1) computing the proximity between nodes; (2) extracting the backbone from the network, represented by a spanning tree, and then partitioning the network based on that backbone; and (3) generating an abstract network. By iteratively applying the same procedures to a newly generated abstract network, we can disclose an abstraction hierarchy implied in a complex network. The Pyramabs flowchart provided in Figure 2 includes the following steps:

Step 1. Input a given network of nodes to Pyramabs.

Step 2. Calculate the proximity between all pairs of nodes and use as the link weights.

Step 3. Normalize the proximity by computing the z-scores; then discard the links with a z-score below a specified threshold to reduce the search space of the network.

Step 4. Obtain the maximum-weight spanning tree from the network and use as the backbone.

Step 5. Partition the network into modules based on its backbone.

Step 6. Construct a network of the modules found in Step 5. This network forms a hierarchical level of a pyramid.

Step 7. If the network produced in Step 6 contains more than one node (i.e., one module), go to Step 2 to find a higher hierarchical level.

Step 8. Otherwise, return the pyramid.

We conducted a series of experiments that applied our approach to datasets from various domains, including artificial and real-world data. Following Sales-Pardo *et al. *[4], we first tested our approach on hierarchically nested random networks with a hierarchical structure. Since the theoretical partition is known in these networks, we can use the results to validate our method's ability to identify the inclusion hierarchy implied in the network. Furthermore, to evaluate the method's generality and its applicability to real-world problems, we tested it on several real-world datasets with different characteristics: protein-protein interactions, metabolic pathways, and social networks [see Additional file 1]. The experimental results indicated that this new method could not only uncover the inherent hierarchy and the significant modules in a complex network, but could also provide different degrees of abstraction of the network.

### Hierarchically nested random networks: inclusion hierarchy

**Figure 3:**
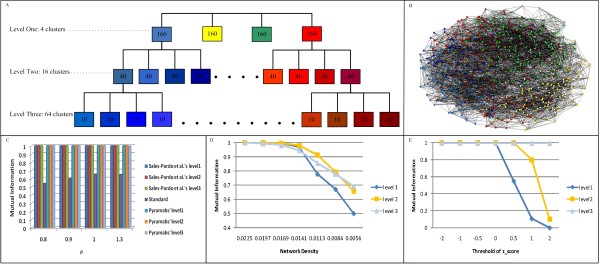
**Validation of two-way module-finding-hierarchy-building strategy**. Pyramabs was validated using 640-node hierarchically nested random networks. (A) The pre-specified nested structures of the random network. (B) An example of a 640-node hierarchically nested random network. Thirty random networks were generated for validation using various values for parameter ρ, which controls the probability that an edge between nodes *i *and *j *exists. The smaller the value of ρ, the more cohesive the levels (see Supporting Information of [4]). (C) The measured accuracy based on the mutual information between the predicted and the real partitions. The accuracy averaged over 30 random networks is provided in the histogram, and shows that Pyramabs was comparable to box clustering, and performed better than the standard module-finding algorithm based on edge betweenness and a modularity measure of the GN algorithm [12,13]. (D) The performance of Pyramabs depending on density. (E) The performance of Pyramabs depending on z-score threshold values.

We verified the ability of Pyramabs to uncover the inclusion hierarchy within an ensemble of random networks proposed by Sales-Pardo *et al. *[4]. There are three levels in the hierarchy, illustrated in Figure 3(A). The top level (level 1) comprises four modules of 160 nodes each. Each top-level module is organized into four sub-modules of 40 nodes each (level 2). Every level-2 module is further divided into four level-3 modules of 10 nodes each. After assigning the nodes to modules, following Sales-Pardo *et al*., we drew an edge between pairs of nodes that have a larger probability of both nodes being assigned to a higher-level module. The probability thus implied a horizontal relationship between nodes. An example random network is shown in Figure 3(B). When tested on 30 randomly generated three-level 640-node artificial networks, Pyramabs performed as well as box clustering [4], and better than a standard module-finding algorithm based on the edge betweenness and modularity measure [12,13] (Figure 3(C)). The density of the random networks tested was 0.0225. To investigate the effect of density on the performance of Pyramabs, we varied the network density by randomly removing links; these results are shown in Figure 3(D). As the density decreased, the mutual information decreased as expected. Nevertheless, Pyramabs could still find the correct hierarchy from the random networks with densities between 0.0225 and 0.0169. The mutual information did not drop noticeably until the graph density fell below 0.0141.

To increase efficiency, Pyramabs reduces the search space using a z-score threshold to filter out "weak" links; the tradeoff is a loss of information. We conducted a series of experiments on random networks, using z-score thresholds ranging from -2 to 2, to evaluate their effect on the results. Pyramabs identified the correct hierarchy with threshold values of -2, -1, -0.5, and 0 (Figure 3(E)). As we further increased the threshold, some supernodes became isolated due to their limited number of links. These experiments illustrated a limitation of Pyramabs that the information loss caused by a low network density or a high z-score threshold has greater influence at the higher levels in the hierarchy, as seen in Figure 3(D) and 3(E). Based on these test results, we set the z-score threshold to zero for the remaining experiments. Because the optimum threshold balancing efficiency and accuracy will vary depending on the network and may not be known beforehand, we made the threshold a user-specified parameter in Pyramabs, with a default value of zero.

### Analysis of a Protein-Protein Interaction Network

We tested Pyramabs on the yeast core protein interaction network previously investigated in [14,15]. The network consists of 2440 proteins connected by 6241 links. As a result of running Pyramabs, we discovered a hierarchy of five abstraction levels. The numbers of modules at each level were 207, 72, 16, 3, and 1; for the 5^th ^(bottom) level to the 1^st ^(top) level, respectively. We evaluated the biological significance of the identified modules based on the Gene Ontology (GO) biological process annotations, using the GO Term Finder of SGD (Saccharomyces Genome Database, http://www.yeastgenome.org/). The GO Term Finder calculates a *p*-value that reflects the probability of observing the chance co-occurrence of proteins with a given GO annotation in a certain module. The smaller the *p*-value, the more consistent the module is with the GO annotations. We used a random assignment of the pool of proteins in the PPI network as the null model. The *p*-value results are presented in Table 1. We included the results of Luo *et al. *[14] and Raddichi *et al. *[15] for reference only, as neither study was capable of extracting a hierarchy from a complex network. To compare the hierarchy detection, we tested the same network using Sales-Pardo *et al*.'s box clustering [4].

**Table 1:** Summary of biological significance of modules based on GO biological process annotations

	*Total Clusters*	***Avg***. *Cluster Size*	***Avg***. *p-value*
**Pyramabs** **(Level 2)**	3	723	2.69E-32

**Pyramabs** **(Level 3)**	16	152	2.59E-20

**Pyramabs** **(Level 4)**	72	34	2.58E-14

**Pyramabs** **(Level 5)**	221	10	5.00E-08

Luo *et al*.^a^	86	19	9.74E-14

Raddichi *et al*.^a^	155	13	3.82E-13

**Sales-Pardo *et al***. **(Level 2)^b^**	77	26	5.45E-14

**Sales-Pardo *et al***. **(Level 3)^b^**	101	11	8.24E-11

**Sales-Pardo *et al***. **(Level 4)^b^**	88	8	6.86E-07

**Sales-Pardo *et al***. **(Level 5)^b^**	12	5	2.12E-04

^a^Both Luo *et al*.'s and Raddichi *et al*.'s methods could only identify single-level modules.

^b^In Sales-Pardo *et al*.'s method, a higher-level module will not necessarily be further partitioned into lower-level sub-modules. Thus, the number of modules does not necessarily increase as the level goes down (e.g., 88 modules at level 4, but only 12 modules at level 5).

From Table 1, it is seen that the average *p*-value decreased at higher levels. This suggested that the vertical relationships in the hierarchy identified by Pyramabs correctly corresponded to the GO hierarchy, since the modules at lower levels correctly merged into larger modules at higher levels. Note that the average cluster sizes at levels 2 and 3 (723 and 152) in our pyramid are much greater than the average level 2 cluster size using box clustering (26). With a closer examination of our level 4 compared with level 3 in box clustering, we found that the total number of clusters was similar (72 vs. 77), as was the average cluster size (34 vs. 26). We further compared these 72 modules with the 77 modules, and found there were a significant number of common module member proteins. The average overlap was over 80%. Based on these findings, Pyramabs was proven to be more useful for disclosing higher-level module organizations than was box clustering. On the other hand, when comparing the bottom level in both hierarchies, our average cluster size was larger (10 vs. 5). This suggests that box clustering has a greater tendency to partition modules into smaller ones than does Pyramabs.

We also analyzed the horizontal relationship in the abstract network at each level to ascertain its ability to characterize biological meaning. The proximity between two supernodes (modules) in an abstract network, as defined in Eq. [3] (see Methods), reflects the significance of the relationship between the nodes. For example, assume there are two pairs of nodes in the abstract network, (*a*, *b*) and (*c*, *d*), for which there is greater proximity between *a *and *b *(denoted by *P_ab_*) than there is between *c *and *d *(denoted by *P_cd_*). Thus, *P_ab _*>*P_cd_*, and *a *and *b *have a closer relationship to each other than *c *and *d*. We assume that if *a *and *b *are related biologically, then the *p*-value calculated by the GO Term Finder will decrease after we merge *a *and *b*. Therefore, if *a *and *b *are closer to each other biologically than *c *is with *d*, then the ratio of decrease in the *p*-value calculated by the GO Term Finder after merging *a *and *b *would be larger than that after merging *c *and *d*. The ratio of decrease in *p*-value is defined as:

$$\begin{array}{l} p-DecreaseRatio(pv_{a},pv_{b})\\ =\frac{\min(pv_{a},pv_{b})-pv_{ab}}{\min(pv_{a},pv_{b})}, \end{array}$$

where *pv_a _*and *pv_b _*are the *p*-values of nodes *a *and *b *calculated by the GO Term Finder, and *pv_ab _*is the *p*-value of the new node consisting of *a *merged with *b*. We used *min*(*pv_a_*, *pv_b_*) in the definition, so a positive *p*-*DecreaseRatio *indicates that *pv_ab _*is smaller than both *pv_a _*and *pv_b_*, i.e., the merge of *a *and *b *is more biologically significant than either *a *or *b*.

In our analysis of protein-protein interactions, we verified whether *a *and *b *actually had a closer biological relationship than *c *and *d *when *P_ab _*>*P_cd_*; this was accomplished by evaluating the change in *p*-value calculated by the GO Term Finder before and after the node (module) merging. We ran a sign test on the abstract network at each level in the hierarchy, and we found a significantly greater number of positive cases in which the ratio of the *p*-value decrease after merging *a *and *b *was larger than that after merging *c *and *d*, when *P_ab _*>*P_cd _*(at the significance level 0.01). These results demonstrated the feasibility of applying a horizontal relationship measured by proximity to the characterization of closeness in biological functions.

**Figure 4:**
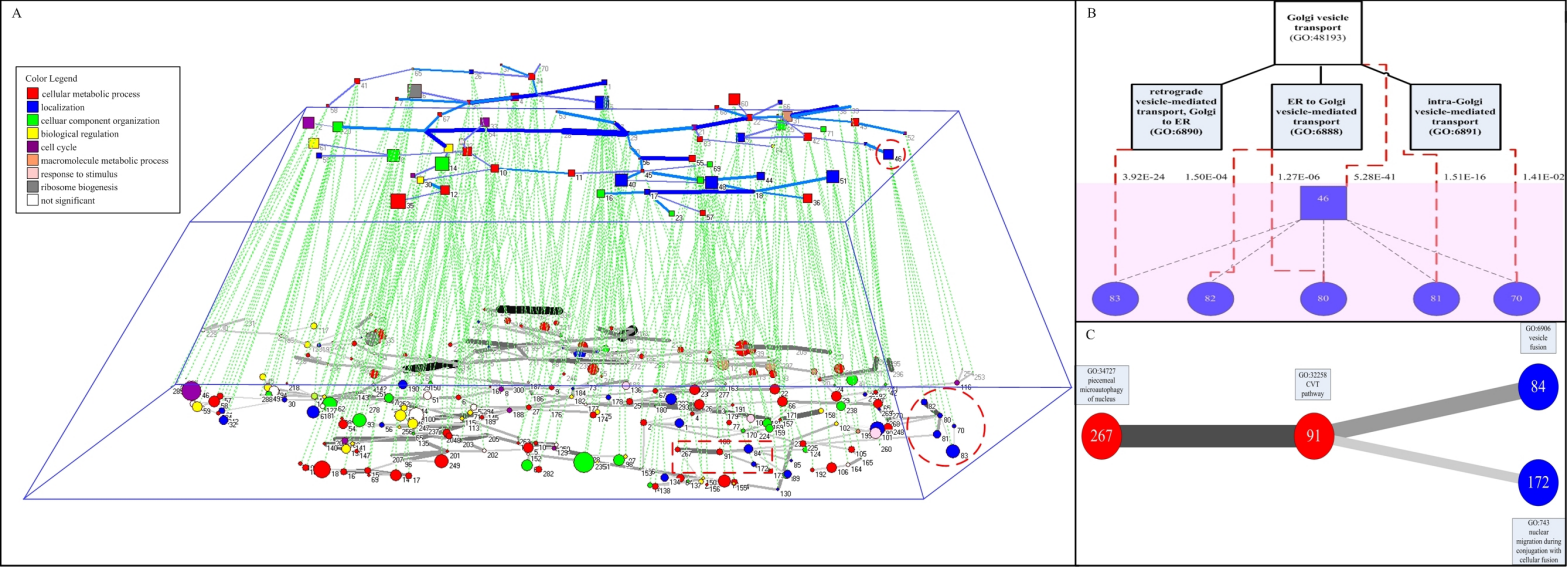
**Vertical relationship between levels 4 and 5, and horizontal relationships among modules at levels 4 and 5 in PPI Network**. (A) The vertical relationship is visualized by dashed green links, and the horizontal relationships at levels 4 and 5 are shown by the solid blue and solid gray links, respectively. With horizontal relationships at the same level, modules enriched by the same annotation category are linked with thicker and darker lines. In contrast, with vertical relationships between different levels, modules enriched by the same annotation category are often merged into a larger module at a higher level. This suggests that Pyramabs can produce a meaningful abstraction hierarchy from the PPI network that corresponds well to known biological processes. (B) The discovered vertical relationships for the five level-5 modules, indexed by 80-83 and 70, which combined to module 46 at level 4. Note that the *p*-value (next to dashed line) for module 46 at level 4 is much smaller than that for each of the modules (80-83 and 70) at level 5. This justified the merge of these modules into a higher-level module. (C) There is greater proximity between modules 267 and 91 than between modules 91 and 84 and between 91 and 172, as indicated by the thickness and darkness of the links. By merging modules 267 and 91 into a new module, we noted a drop in *p*-value by 49.49%. Compared with combining modules 91 and 84 (19.76% drop in *p*-value) or modules 91 and 172 (3.17% drop in *p*-value), the greater proximity between modules 267 and 91 in the horizontal relationship correctly demonstrates their greater functional similarity.

Due to the complexity of Figure 4(A), we show the backbone of the abstract network at levels 4 and 5 in the hierarchy instead of the complete abstract networks. The biological meaning behind the pyramid structure represents how these proteins relate and interact at different hierarchical levels. One example, marked by two red circles of the vertical relationship, was selected for further study, and involved one level-4 module and five level-5 modules, as detailed in Figure 4(B). We also selected one example of the horizontal relationship for further analysis, marked by a red rectangle. This included four level-5 modules, described in Figure 4(C). The vertical relationships corresponded correctly to the GO hierarchy, and the strength of the horizontal relationships provides a good indication of the functional closeness between protein modules.

### Analysis of a Metabolic Network

Thousands of components in a living cell are dynamically interconnected within a complex network that determines the cell's functional properties [5,16]. One of the primary examples is cellular metabolism arising from sophisticated biochemical networks, in which numerous metabolites are integrated through biochemical reactions. To facilitate the identification and characterization of system-level features in biological organizations, we can partition cellular functionality into a collection of modules and organize them in a hierarchy [11]. We tested Pyramabs on the metabolic network of *E. coli *that was used previously [4]. This network contained 507 nodes and 947 links, where each node represented a metabolic substrate, and each link described a reaction.

In KEGG [17], metabolic pathways are classified into 11 categories: Carbohydrate, Energy, Lipid, Nucleotide, Amino acid, Other amino acid, Glycan, PK/NRP, Cofactor/vitamin, Secondary metabolite, and Xenobiotics. Each category consists of several sub-categories (e.g., nucleotide metabolism includes purine metabolism and pyrimidine metabolism). In addition to the KEGG PATHWAY classifications, KEGG also provides Pathway Modules that are specifications of sub-networks corresponding to tighter functional units. Following previous work [18-20], we evaluated the biological relevance of a module by conducting an enrichment analysis based on the KEGG annotation categories. Likewise, we used the hypergeometric distribution to obtain a *p*-value for the fraction of metabolites in each module associated with an annotation category of KEGG; this was used to measure the within-module consistency of metabolic pathway classification. The *p*-values were calculated according to the KEGG PATHWAY category, PATHWAY sub-category, and Pathway Module, respectively. The results are summarized in Table 2. For comparison, we tested the same network using box clustering [4], with results shown in Table 3. From these results, we noted that both the total number of clusters and the average cluster size were similar between the Pyramabs level 3 and the box clustering level 2 (26 vs. 28 for the number of clusters, and 20 vs. 18 for the average cluster size). However, the average cluster size in the Pyramabs level 2 was much larger than that in the box clustering level 2 (85 vs. 18). This demonstrated that Pyramabs identified one additional higher level in the module organization than did box clustering. We also observed that the average cluster size for levels 3, 4, and 5 for the box clustering was smaller than that for Pyramabs' bottom level (3 vs. 5). Similar to the findings in the PPI network, these results suggest that box clustering is more likely to produce organizations of smaller modules in the hierarchy compared with Pyramabs. This analysis suggests the possibility of combining the two complementary approaches to identify more detailed module organizations.

**Table 2:** Summary of the within-module consistency of metabolic pathway classification by Pyramabs based on KEGG

	*Total Clusters*	***Avg***. *Cluster Size*	*PATHWAY Category* *Avg. p-value*	*PATHWAY* *Sub-category* *Avg. p-value*	*Pathway* *Module* *Avg. p-value*
**Level 2**	6	85	4.77E-12	4.79E-15	1.57E-07

**Level 3**	26	20	4.27E-07	4.71E-12	4.15E-08

**Level 4**	104	5	2.06E-03	3.68E-06	4.79E-05

We do not show level 1, which has only one cluster.

**Table 3:** Summary of the within-module consistency of metabolic pathway classification by Sales-Pardo *et al*.'s box clustering based on KEGG

	*Total Clusters*	***Avg***. *Cluster* *Size*	*PATHWAY Category* *Avg. p-value*	*PATHWAY* *Sub-category* *Avg. p-value*	*Pathway* *Module* *Avg. p-value*
**Level 2**	28	18	5.67E-06	1.41E-10	7.37E-07

**Level 3**	63	6	1.34E-03	3.86E-06	8.03E-05

**Level 4**	48	3	1.96E-02	5.60E-04	3.20E-03

**Level 5**	50	3	2.21E-02	7.14E-04	3.00E-03

We do not show level 1, which has only one cluster.

**Figure 5:**
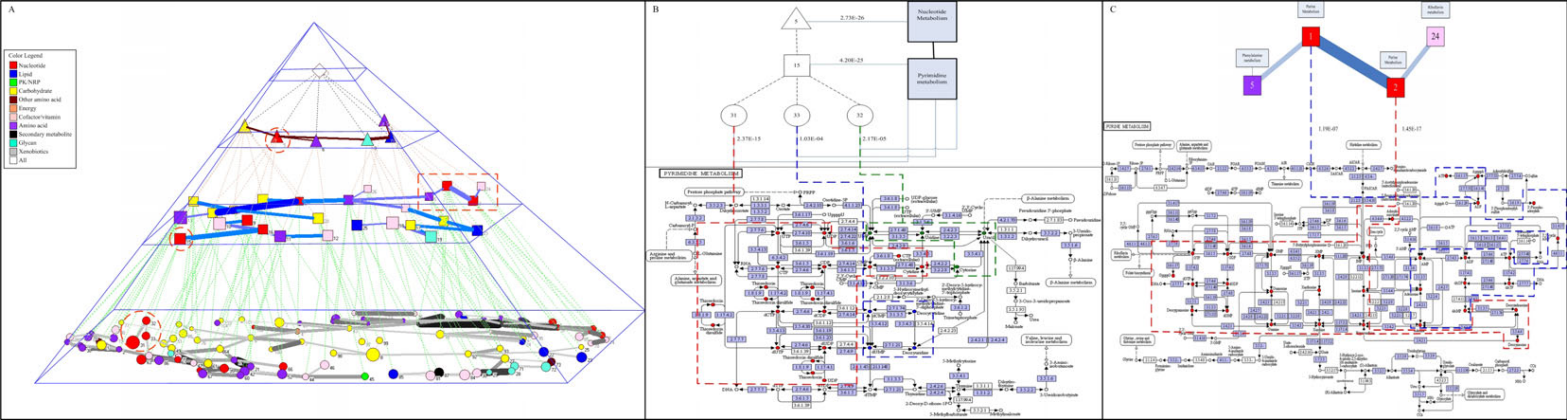
**Abstraction pyramid disclosed from metabolic network**. (A) Modules denoted by different shapes at each level, with shape sizes representing the relative module sizes. Vertical relationships between two levels are visualized by black, pink, and green dashed lines. Horizontal relationships are shown by solid brown, blue, and gray lines at the 2^nd^, 3^rd^, and 4^th ^levels, respectively. (B) Modules 31, 32, and 33 at level 4 were most consistent with "Pyrimidine metabolism," a sub-category of the KEGG PATHWAY "Nucleotide metabolism," with *p*-values of 2.37E-15, 2.17E-05, and 1.03E-04, respectively. We marked the modules with the red, green, and blue dashed lines on the pyrimidine metabolism map provided in KEGG. Merging modules 31, 32, and 33 into a larger module 15 showed higher within-module consistency with the Pyrimidine metabolism sub-category (*p*-value = 4.20E-25). Module 15, combined with others, formed a level-2 module 5, which mapped most consistently onto the "Nucleotide metabolism" metabolism pathway category, with a *p*-value of 2.73E-26. These results demonstrated that the vertical relationships disclosed by Pyramabs could characterize *inclusion *(or *part-of*) relationships, e.g. "Pyrimidine metabolism" is a sub-category of "Nucleotide metabolism." (C) Modules 1 and 2 are marked with the blue dashed line and the red dashed line on the purine metabolism map in KEGG. The remaining modules in the horizontal relationship mapped onto different pathway categories. The greatest proximity in the horizontal relationship occurred between modules 1 and 2. This result is consistent with modules 1 and 2 belonging to the same metabolism pathway category, while the others belong to various other categories.

Figure 5(A) shows the abstraction pyramid extracted from the metabolic network. To enhance readability, we only presented the spanning tree of the abstract network at each level. An example of the vertical relationship between different hierarchical levels was marked by red circles for further analysis (Figure 5(B)). We also used a red rectangle to highlight an example of the horizontal relationship at the 2^nd ^level, and compared it against the KEGG PATHWAY (Figure 5(C)). The vertical relationships disclosed by Pyramabs correctly characterized *inclusion *(or *part-of*) relationships (e.g., "Pyrimidine metabolism" is included in "Nucleotide metabolism"); the horizontal relationships showed that the modules with greater proximity between them are more likely to belong to the same pathway category.

## Discussion

One widely used method for finding the organization within data is hierarchical clustering [11,21,22]. Hierarchical clustering techniques group data into a sequence of nested clusters, either by treating each singleton as a cluster and merging them into larger clusters (agglomerative or "bottom-up"), or by dividing an initial single cluster into successively smaller clusters (divisive or "top-down"). Both techniques organize data into a hierarchical structure, typically depicted as a dendrogram. Both agglomerative and divisive clustering techniques produce a hierarchical tree allowing the visualization of the internal hierarchical structure within data, regardless of whether or not the data are actually organized hierarchically. It can be argued that a "height threshold" in a dendrogram can be judiciously selected according to some metric, above which any clusters and their hierarchical relationships are regarded as genuine. Nevertheless, it is debatable if any post-clustering analysis that is independent of the clustering process will be effective. Box clustering has been proposed as a variant of divisive unsupervised clustering [4]. This method iteratively identifies the modules at each level in the hierarchy until no further hierarchical levels can be found through module division. Although it visualizes the final clustering result by a box-model clustering tree, it only shows vertical relationships between different hierarchical levels.

The quality of the network interpretation depends on the completeness of the knowledge extracted and the expressiveness of the knowledge representations. The present paper provides two contributions to this area. First, we proposed the abstraction pyramid, a new representation that combined vertical and horizontal viewpoints and is capable of interpreting a complex biological network at different degrees of abstraction. Interpretations in this form are more accurate and more meaningful than multilevel dendrograms or single-level graphs. Second, we developed Pyramabs, a two-way approach combining top-down and bottom-up clustering techniques to detect modules and organize them into a multilevel pyramid. As an improvement, the abstraction pyramid gives us the opportunity to achieve a new perspective on cellular organization, by traversing the pyramid freely through the links vertically and horizontally. For example, in one pyramid, we can learn how the metabolites in the metabolic pathways at the bottom level are merged into functional modules through vertical links. We can also verify if the higher-level modules connected by the horizontal links show any topological property, e.g., scale-free connectivity, that is shared by natural and social networks [11,23]. With a macro view, we can investigate the changes in topological properties and the biological meanings from one abstraction level to another. In contrast, with a micro view, we can analyze all possible routes going through the modules across levels, to identify interesting attributes or patterns. The modularity and hierarchy concepts have long been popular in various fields, e.g., biology, psychology, sociology, and digital system design [21,24-26]. Our abstraction pyramid combines these two concepts. It allows the details of each module to be dealt with in isolation, or the overall characteristics of a coherent system to be dealt with at different levels. This integrated concept is similar to a computer architecture design or system engineering, in which computing modules are organized in a hierarchy according to functionality and implementation details. We expect that future studies in these directions will shed light on new research topics within these fields.

## Conclusions

To evaluate the interpretations made on complex biological networks by Pyramabs, we experimented on PPI and metabolic networks. The experiments showed that the abstraction pyramids were biologically meaningful. The vertical relationships successfully characterized the *inclusion *relationship according to the GO and KEGG category hierarchy, and the strength of the horizontal relationships correctly reflected the functional closeness according to the GO and KEGG annotations. In addition, we tested Pyramabs on two social networks to demonstrate its generality: Zachary's karate club network and an NCAA college football network [see Additional file 1]. These results were encouraging.

We can extend this work in several directions. One future improvement of Pyramabs is to identify overlapping modules. Currently the modules at the same level are not allowed to overlap, although overlapping modules exist in some real-world domains. Second, although the performance of Pyramabs has been demonstrated in real-world domains, we can refine the proximity measure and utilize domain knowledge to improve its robustness for situations in which networks may contain spurious links and nodes, or may be missing crucial links or nodes. Pyramabs currently assumes that the given network is correct when it extracts the abstraction pyramid from the complex network. Third, we can characterize an algorithm for network community analysis, using the proximity measure applied to evaluate the association between nodes, and using the construction procedure it takes to organize the communities. A more thorough comparative study of Pyramabs with other methods provides the opportunity to integrate various complementary algorithms to increase its applicability to various domains and its accuracy in interpreting the networks.

## Methods

### Proximity Measure

There is great flexibility in how we define the proximity between a pair of nodes, and the selection of an appropriate proximity function is crucial since it will affect the formation of the resulting modules. Several measures are commonly used, including Euclidean distance, correlation coefficient, and cosine similarity [22,27]. Here, we investigated the use of clustering based on network topology alone. Conventional proximity measures are not applicable to clustering problems if the network topology is the only information given (e.g., we cannot calculate Euclidean distance without the node coordinates). Other proximity measures, such as edge betweenness [12,13,21] and topological overlap [11,28,29], were recently proposed and used in the study of social, metabolic, protein-protein interaction, and gene networks. In spite of having some successful applications, they have limitations. The edge betweenness of a pair of nodes reflects the global characteristics of a network, but suffers from high computational cost [13,15,21] and the effects of incompleteness and noise in the network [14,30]. The topological overlap is a local measure, and may fail to identify any module beyond a locally dense connectivity pattern [4].

Most of the current proximity measures do not account for link direction or link weight. We propose a new proximity function with expanded applicability that handles link directions and weights. For simplicity, we describe a directed weighted network of *n *nodes by an *n *× *n *adjacency matrix *A*, in which each element *A_ij _*is the weight of the link from node *i *to *j*. A zero valued weight (*A_ij _*= 0) indicates no link from node *i *to *j*. We define the proximity function *prox*(*i*,*j*) from node *i *to *j*, *i *≠ *j*, as

(1) $$\begin{array}{l} prox(i,j)\\ =A_{ij}\\ +\sum_{k}^{A_{ik}\neq 0,A_{kj}\neq 0}\left\{\frac{A_{ik}}{W_{i}^{out}-A_{ij}}\times \frac{A_{kj}}{W_{k}^{out}}\times \min(Aik,Akj)\right\}\\ W_{i}^{out}=\sum_{m}^{n}Aim \end{array}$$

where $W_{i}^{out}$ is the sum of the weights of all outgoing links of node *i*. Our proximity function considers not only the effects of common neighbors (i.e., node *k*), but also the link direction and the link weight. According to studies of protein-protein interaction [31-33], often two interacting proteins share no functional pathways, but reveal substantial functional similarity to their common neighbors. These observations suggest that we treat direct links and indirect paths differently. We assume that the weight of the direct link, *A_ij_*, directly contributes to the proximity, *prox*(*i*,*j*), as indicated by the first term in Eq. [1].

On the other hand, to calculate the proximity between *i *to *j *based on an indirect path from *i *to *j *by way of *k*, we divide the path into two sub-paths, *i *to *k *and *k *to *j*. Unlike direct links, we hypothesize that on an indirect path, one node does not always affect all its neighbors; rather, it acts probabilistically. For an indirect path from *i *to *j *by way of *k*, the probability that node *i *affects node *k *is defined as the ratio of the link weight between *i *and *k *to the sum of the weights of all outgoing links of node *i*, except the direct link from *i *to *j*. The probability that node *k *affects node *j *is defined as the ratio of the link weight between *k *and *j *to the sum of the weights of all outgoing links of node *k*. The probability of the complete indirect path from *i *to *j *by way of *k *is then the product of the probabilities of the path from *i *to *k *and the path from *k *to *j*. The proximity contributed by the indirect path from *i *to *j *by way of *k *is determined by both the probability of the indirect path and the link weights *A_ik _*and *A_kj_*. If there is more than one common neighbor of *i *and *j*, we sum the proximity of each indirect path, as shown in the second term in Eq. [1]. Although the proposed proximity function is a local measure, like topological overlap, it has better discrimination in network topology [see Additional file 2], and requires less computational effort than a global measure (e.g., edge betweenness). Incorporating the proximity function into a two-way module-finding-hierarchy-building strategy, we can gather the local and global characteristics, and detect the hierarchical structure of the network.

### Extracting Network Backbone and Partitioning Network

The optimal solution to the partition of a network, based on some criterion function, can be found by enumerating all possibilities. However, this is computationally prohibitive for large practical networks. To reduce the problem space, we adopted a graph-theoretic approach to partitioning [34]. After computing the proximity between all pairs of nodes, we build a maximum spanning tree [35] that includes all the nodes of the network, and connect these nodes with the maximum sum of the link proximity. We view the maximum spanning tree as the backbone of the network, and discard the links with less significant proximities. We perform partitioning based on the maximum spanning tree, rather than the original network, in order to reduce computational cost.

We obtain two sub-trees by removing one link from a tree; each sub-tree then represents a module. By repeating this process on each sub-tree in a top-down fashion, we can partition a tree into many sub-trees (i.e., modules/clusters). Given the maximum spanning tree, we examine the modules created by iteratively removing one link from a (sub)tree. A link is selected for removal if, after removing the link, the set of the modules *M *= {*M_1_*,*M_2_*,*M_3_*,...,*M_n_*} meets the following criteria:

(2) $$\begin{array}{l} \forall M_{a},M_{b}\in M,\;a\neq b\;\\ S_{\mathrm{int}ra}^{Ma}>S_{\mathrm{int}er}^{Ma,Mb}\text{ and }S_{\mathrm{int}ra}^{Mb}>S_{\mathrm{int}er}^{Ma,Mb} \end{array}$$

where $S_{\mathrm{int}ra}^{M_{k}}=\sum_{\forall i,j\in M_{k}}A_{ij}$ is the sum of the proximity of each intralink within *M_k_*, and $S_{\mathrm{int}er}^{Ma,Mb}=\sum_{\forall i\in M_{a},j\in M_{b}}A_{ij}$ is the sum of the proximity of each interlink between *M_a _*and *M_b_*.

Our criteria for modules are similar to those previously proposed [14,15], but with a focus on the link weight (proximity) instead of on the degrees of nodes. Note that the sum of proximity in Eq. [2] is calculated on the network rather than on the tree, to avoid information loss. We use the tree only for evaluating which nodes are clustered to reduce the search space of the original network. We provide the pseudocode for the top-down network partitioning procedure [see Additional file 3].

### Network Abstraction

After partitioning the network, we treat each module as a supernode [36]. The supernodes network is viewed as an abstraction of the original network, and it reveals the general framework of the original network without the loss of its principal characteristics. We define the proximity between a pair of supernodes (e.g., modules *M_a _*and *M_b_*) as follows:

(3) $$\begin{array}{l} prox_{\sup er}(M_{a},M_{b})\\ =\frac{1}{\left|M_{a}\right|\cdot \left|M_{b}\right|}\sum_{\forall m\in M_{a},n\in M_{b}}prox(m,n) \end{array}$$

where |M_a_| is the number of nodes in module M_a_. We first compute the proximity between all possible pairs of supernodes, and then normalize the proximity to a z-score. Those links with a z-score below a threshold (currently set to zero) are considered insignificant, and thus discarded from the network. The resulting network of supernodes is the abstraction of the original network, and is placed one level higher than the original network in the hierarchy. By repeating this process on the networks in the hierarchy, we can generate additional abstract networks and continue building the pyramid of abstraction consistently and systematically from the bottom up, as illustrated in Figure 1.

## Authors' contributions

CYC initiated the concept of network abstraction, implemented Pyramabs, and conducted the experiments. YJH designed the proximity measure and the pyramid building procedure, and supervised this study. Both authors read and approved the final manuscript.

## Supplementary Material

Additional file 1 **Analysis of social networks**. The data provided represent the analysis of two social networks, Zachary's karate club network and NCAA football game network. Click here for file

Additional file 2 **Comparison of proximity function and topological overlap measure**. The data provided represent the examples that show the difference between our proximity function and previous topological overlap measure. Click here for file

Additional file 3 **Pseudocode of network partition**. The data provided represent the pseudocode of our top-down network partition. Click here for file
