# Supplementary material for: Extracting the abstraction pyramid from complex networks
Source: BMC Bioinformatics. 2010 Aug 3;11:411. doi: 10.1186/1471-2105-11-411 (PMC2921411; doi:10.1186/1471-2105-11-411)
Supplement: Additional file 1 — Analysis of social networks. The data provided represent the analysis of two social networks, Zachary's karate club network and NCAA football game network. [file 1471-2105-11-411-S1.DOC]

**Analysis of social networks**

**Analysis of Social networks: Zachary’s karate club network and NCAA football game network**

We selected two social networks to test Pyramabs. The module structures of these networks are known. The first was Zachary’s karate club network (Figure S1A), which consisted of 34 nodes and 78 edges [1] representing the pattern of friendships among the members of a karate club at a U.S. university, over a period of two years in the early 1970s. The club was known to split into two groups, administrators and instructors. A previous method [2, 3] found a partition in agreement with the actual split, except that node 3 was misclassified. With the Pyramabs test, the karate club network was partitioned into two modules corresponding to the actual factions found in Zachary’s study, as shown in Figure S1B.

We next ran Pyramabs on a NCAA college football network consisting of 115 nodes and 613 links [2]; this network represented the schedule of Division I games between American college football teams during the year 2000 season. The teams were organized into different conferences, with intra-conference games scheduled more frequently than inter-conference games. We detected an abstraction pyramid with three hierarchical levels. The 12 communities that Pyramabs identified at the bottom level (Figure S2A) mapped well to the 12 college football conferences. Nearly all of the teams were clustered correctly with other members in the same conference. The independent teams that were scattered among several communities reflected the fact that these teams did not belong to any conference. The results shown in Figure S2A were comparable to those previously reported [2]. Figure S2B shows the abstract network of the communities detected from the original football game network. Unlike previous studies, from the bottom-level abstract network, Pyramabs further identified two modules (Figure S2C) that were separated geographically by the Mississippi River (Figure S2D).

**References**

1. Zachary, W.W. (1977) Information-flow model for conflict and fission in small-groups. *J Anthropol Res* 33:452-473.

2. Girvan, M. and Newman, M.E.J. (2002) Community structure in social and biological networks. *Proc Natl Acad Sci USA* 99:7821-7826.

3. Newman, M.E.J. and Girvan, M. (2004) Finding and evaluating community structure in networks. *Phys Rev E* 69.

**Figures**

**Figure S1 - Clustering results for Zachary’s karate club network**

**
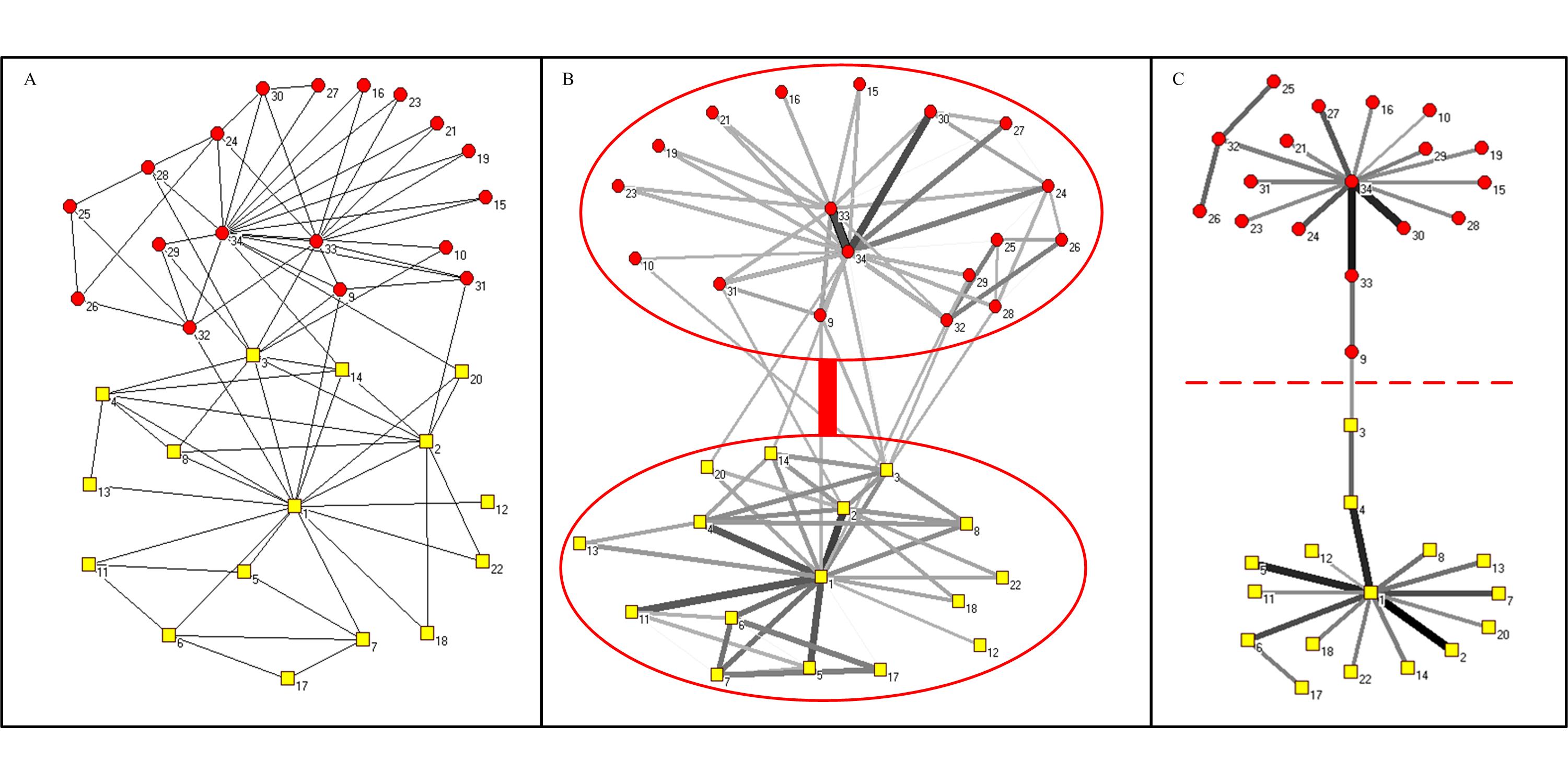
**

(A) Zachary’s karate club network, consisting of 34 nodes and 78 edges. The nodes (i.e., club members) were categorized into two groups: administrators and instructors, shown by red circles and yellow squares, respectively. (B) The proximity network for Zachary’s karate club. A thicker, darker link between nodes indicates a closer proximity. (C) The backbone, represented by a spanning tree, extracted from the proximity network. Pyramabs identified the correct cut between node 3 and node 9. Based on the tree and the cut line, we identified two clusters, as shown in (B), with two red circles connected by a thick red link. The abstract network of the two modules corresponds to the correct partition for the karate club.

**Figure S2 - Clustering results for an American college football game network**

**
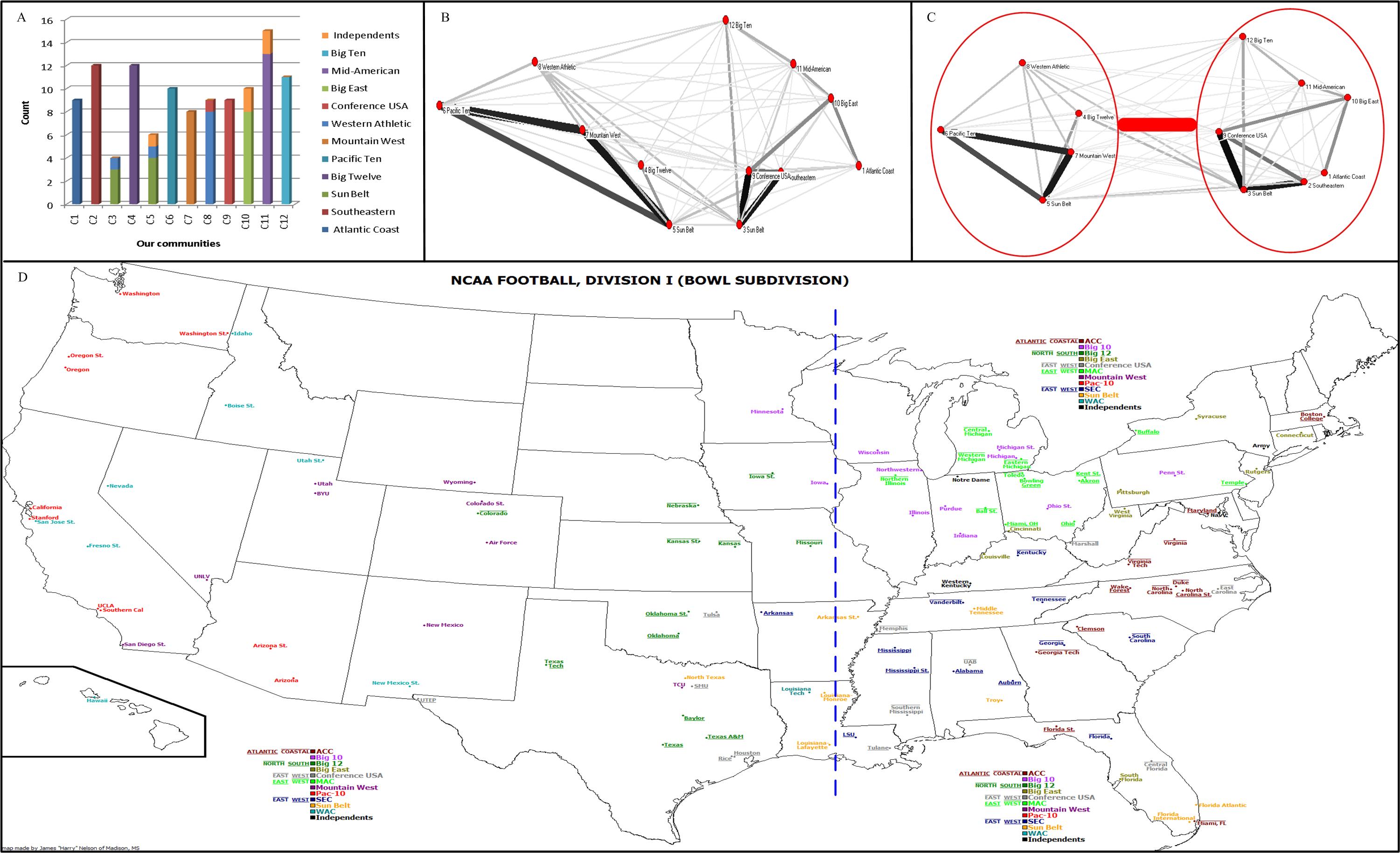
**

(A) Twelve communities identified by Pyramabs. Similar to previous results [2], the communities mapped well to the 12 NCAA conferences, except for a few cases that were due to nuances in the scheduling of games (e.g., the SunBelt teams). The independent teams were distributed among several conferences, demonstrating that they did not belong to any conference. (B) The abstract network of the 12 communities showing the proximity between communities. The thicker and darker the link, the closer the pair of communities to each other. (C) The second-layer abstract network represented by red circles connected by a red line. Five conferences were grouped in one module; the remaining seven were in the other module. (D) The conferences placed on the map. Interestingly, two modules on the second abstraction layer were separated geographically by the Mississippi River (blue dashed line).
