# Supplementary material for: Extracting the abstraction pyramid from complex networks
Source: BMC Bioinformatics. 2010 Aug 3;11:411. doi: 10.1186/1471-2105-11-411 (PMC2921411; doi:10.1186/1471-2105-11-411)
Supplement: Additional file 2 — Comparison of proximity function and topological overlap measure. The data provided represent the examples that show the difference between our proximity function and previous topological overlap measure. [file 1471-2105-11-411-S2.DOC]

**Comparison of proximity function and topological overlap measure**

We used the examples shown in Figure S3 to illustrate our proximity function, and compared it with a related measure, topological overlap, as both take common neighbors into consideration. The topological overlap measure *T_ij_* between node *i* and node *j*, *i* ≠ *j*, is defined as

$$T_{ij}=\frac{l_{ij}+a_{ij}}{\min(d_{i},d_{j})+1-a_{ij}}$$

where *l_ij_* is the number of common neighbors of node *i* and node *j*, *d_i_* is the degree of node *i*, and *a_ij_*=1 if there exists a direct link between *i* and *j*; otherwise, *a_ij_*=0. The term 1- *a_ij_* is needed in the denominator to prevent the denominator from becoming zero if min(*d_i_*,*d_j_*)=0. The inclusion of *a_ij_* in the numerator makes *T_ij_* explicitly dependent on the direct link between *i* and *j.* These examples demonstrate that our proximity function has better discrimination in network topology than the topological overlap measure.

**Figure S3 - Four simple networks to illustrate proximity measures**

**
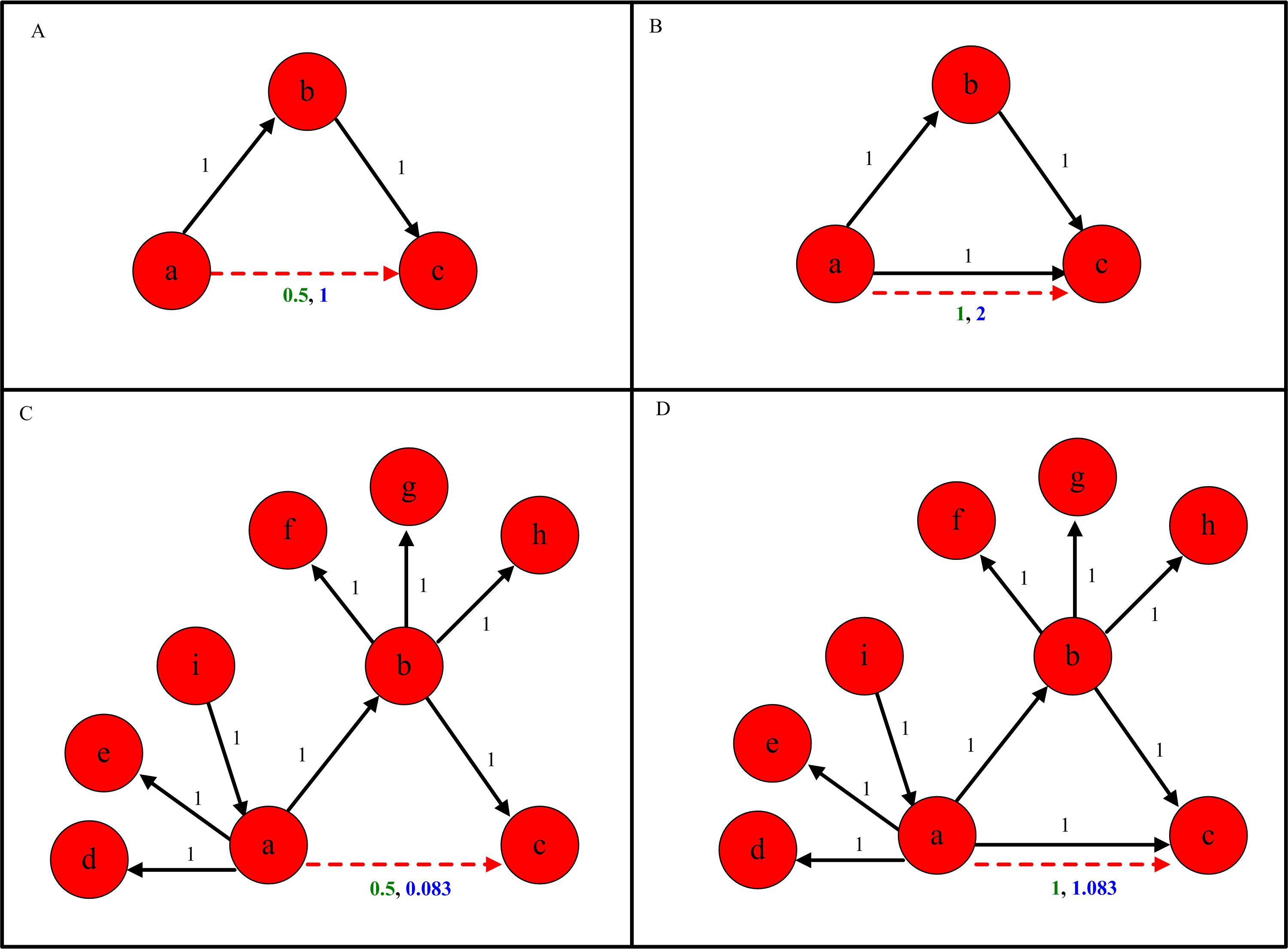
**

We compared our proximity function with a topological overlap measure. Link weights are given by the number next to a black link. For simplicity, the weights were all set to 1 without loss of generality. We calculated the proximity between node *a* and node *c*, using our proximity function and the topological overlap measure. The dashed red link represents the proximity accompanied by two proximity values. The blue number is the proximity calculated by our function; the green number is from the topological overlap measure. (A) A simple three-node network without a direct link from node *a* to node *c*. (B) Unlike the previous example, there exists a direct link from node *a* to node *c* in this network. According to the two measures, both proximity values increase as expected, where *T_ac_*=1 and *prox*(*a*,*c*)=2. (C) We made a larger network from the first three-node network by adding more neighbors to nodes *a* and *b*. The topological overlap measure gives the same proximity value, 0.5, between node *a* and node *c* as in (A). However, the proximity function we propose returns a lower value, 1/12 (i.e., 1/3×1/4×1=1/12), which reflects the change in network topology. (D) After adding the direct link between nodes *a* and *c*, the proximity computed by either measure increases as expected.
