# Supplementary material for: Extracting the abstraction pyramid from complex networks
Source: BMC Bioinformatics. 2010 Aug 3;11:411. doi: 10.1186/1471-2105-11-411 (PMC2921411; doi:10.1186/1471-2105-11-411)
Supplement: Additional file 3 — Pseudocode of network partition. The data provided represent the pseudocode of our top-down network partition. [file 1471-2105-11-411-S3.DOC]

**Pseudocode of network partition**

Starting with the single module represented by the maximum spanning tree, we show the pseudocode of the partition procedure below. The input includes the network in question, *Net*, and its maximum spanning tree, *M_1_*. The output contains the clusters of the partitioning result.

Procedure Network_Partition (*Net*, *M_1_*)

*M*={*M_1_*} //*M* keeps the modules for further analysis

Repeat

{

Select largest cluster *M_i_* ∈ *M*, and remove *M_i_* from *M*.

Put *M_i_* into *D*. //*D* stores the final clusters

Put all the links of *M_i_* in *L_i_*.

While (*L_i_* is not empty)

{

Set the link in *L_i_* with min proximity as *l_min_*.

Remove *l_min_* from *L_i_*. //If there’s more than one *l_min_*, remove one randomly

Generate two modules (i.e. sub-trees) *M_a_* and *M_b_* by removing *l_min_* from *M_i_*.

Add *M_a_* and *M_b_* to *M*.

If *M* does not satisfy module criteria

{

Remove *M_a_* and *M_b_* from *M*.

Restore *l_min_* to *M_i_*. //put the link *l_min_* back to the tree *M_i_*

}

Else

{

Remove *M_i_* from *D*. //because *M_i_* is legally split into *M_a_* and *M_b_*

Break; //break out of While loop

}

}

} until *M* is empty.

Output *D*.
